# Complete mitochondrial genomesequence of the hen harrier (*Circus cyaneus*)

**DOI:** 10.1080/23802359.2018.1481783

**Published:** 2018-06-07

**Authors:** Xiaodong Gao, Guolei Sun, Tian Xia, Chao Zhao, Qinguo Wei, Weilai Sha, Honghai Zhang

**Affiliations:** College of Life Science, Qufu Normal University, Qufu, P.R. China

**Keywords:** Mitochondrial genome, circus cyaneus, phylogenetic analysis

## Abstract

In this study, the complete mitochondrial genome of the Hen Harrier (*Circus cyaneus*) was sequenced. The genome is found to be 18,754 bp in length and has a base composition of A (31.7%), G (13.0%), C (31.2%), and T (24.1%). Similar to other *Circus* species, it contains a typically conserved structure including 13 protein-coding genes, 2 rRNA genes, 2 control region (D-loop), and 22 tRNA genes. A phylogenetic analysis between 17 species using 12 protein-coding genes proved that *Accipiter gentilis* is the most close to *Circus cyaneus.*

In this study, the complete mitochondrial genome of the Hen Harrier (*Circus cyaneus*) was sequenced and reported for the first time using muscle tissue obtained from a wild individual in Hulun Lake National Nature Reserve in Inner Mongolia, China (48.58°N, 117.65°E).

The Hen Harrieris a raptorial bird belonging to the family Accipitridae, it is a small migrant raptor with a wide breeding range from Europe and North Asia to Russian Far East. Winters from Europe and Northwest Africa through Turkey and Middle East to Southeast China, Korea and Japan (Ferguson-Lees and Christie 2006).

The complete mitochondrial genome sequence of the Hen Harrier was deposited in GenBank after accurately annotated with the accession number KX925606. Its total length is 18,754 bp and sequence analysis showed its structure is similar to the other raptors, which contains two rRNA genes, 22 tRNA genes, 13 protein-coding genes, two control region. The total composition is 33.2% C, 30.9% A, 22.8% T and 13.1% G, and a strong A-T bias (53.7%) is found, The gene content, structure, and arrangement of the *Circus cyaneus* are similar to other Accipitridae (Haring et al., 2001; Li et al., 2015; Dou et al., 2016).

Thirty genes are encoded on the heavy strand, while nine genes (ND6 and 8 tRNA genes) on the light strand. Start codons included ATG (11 PCGs), GTG (one PCG), and ATC (one PCG), while stop codons include TAA (seven PCGs), T–(three PCGs), AGG (one PCG), TAT (one PCG), TAG (one PCG). The 16S rRNA and 12S rRNA were 1601 and 980 bp long respectively. The Hen Harrier mitochondrial genome contains two large non-coding regions. The first control region (D-loop) is 2,248 bp in length, located between tRNA^Thr^ and tRNA^Pro^, and the second is 935 bp, located behind tRNA^Glu^.

To analyse the phylogenetic relationships between *Circus cyaneus* and other species, we used PAUP 4.0 and MrBayes 3.2.3 (Tamura et al. 2013) to perform phylogenetic analyses based on 12 protein-coding genes(except ND6 gene) with 16 species derived from the order of Accipitriformes and Falconiformes, *Phasianus colchicus*was used as outgroup. *Circus cyaneus* was most closely related to *Accipiter* (Figure 1). The evolutionary relationships of these analysed species are consistent with previously reported results (Lerner & Mindell, 2005). The newly characterized mt genome will help to understand the evolution of Harriers.

**Figure 1.**
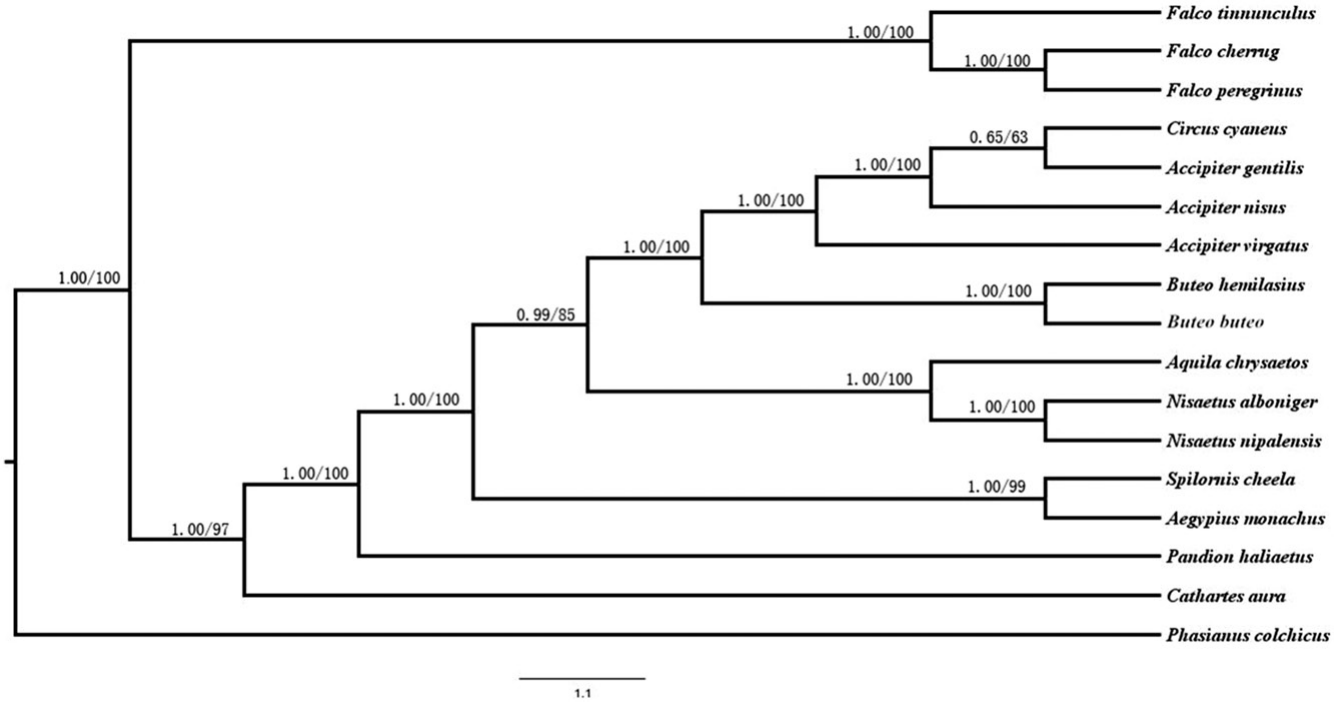
Maximum likelihood (ML) and Bayesian inference (BI) tree based on complete chloroplast genome sequences of 17 species using *Phasianus colchicus* as an outgroup. All 17 species’s accession numbers are listed as below: *Circus cyaneus* KX925606, *Accipiter gentilis* NC_011818, *Accipiter nisus* NC_025580, *Accipiter virgatus* NC_026082, *Buteo emilasius* NC_029377, *Buteo buteo* NC_003128, *Aquila chrysaetos* NC_024087, N*isaetus alboniger* NC_007599, *Spilornis cheela* NC_015887, *Nisaetus nipalensis* NC_007598, *Pandion haliaetus* NC_008550, *Aegypius monachus* KF682364, *Cathartes aura* NC_007628, *Falco tinnunculus* NC_011307, *Falco cherrug* NC_026715, *Falco peregrinus* NC_000878, *Phasianus colchicus* NC_015526.
